# In vitro radiosensitivity of tumour cells and fibroblasts derived from head and neck carcinomas: mutual relationship and correlation with clinical data

**DOI:** 10.1038/sj.bjc.6690172

**Published:** 1999-03

**Authors:** B Stausbøl-Grøn, S M Bentzen, K E Jørgensen, O S Nielsen, T Bundgaard, J Overgaard

**Affiliations:** Danish Cancer Society, Department of Experimental Clinical Oncology, Aarhus University Hospital, Denmark; Department of Ear, Nose and Throat Diseases, Odense University Hospital, Denmark; Department of Oncology, Aarhus University Hospital, Denmark; Department of Ear, Nose and Throat Diseases, Aarhus University Hospital, Denmark

**Keywords:** predictive assays, in vitro radiosensitivity, squamous cell carcinoma of the head and neck, differentiation, fibroblasts, tumour cells

## Abstract

The aim was to characterize the variation in the cellular in vitro radiosensitivities in squamous cell carcinomas of the head and neck, and to test for a possible correlation between different measures of radiosensitivity and the clinical and histopathological data. Cellular in vitro radiosensitivities were assessed in tumour biopsies from 71 patients using the modified Courtenay–Mills soft agar clonogenic assay combined with an immunocytochemical analysis. Radiosensitivity was quantified as the surviving fraction after a radiation dose of 2 Gy irrespective of cell type (overall SF_2_), or based on identification of cell type (tumour cell SF_2_, fibroblast SF_2_). Sixty-three biopsies were from primary tumours, and eight were from recurrences. Overall plating efficiency ranged from 0.005 to 1.60% with a median of 0.052%. The majority of the colonies obtained from the biopsies were fibroblast marker-positive; the proportion of tumour marker-positive colonies ranged from 1 to 88% with a median of 15%. The median overall SF_2_ was 0.47 (range 0.24–0.96), the median tumour cell SF_2_ was 0.50 (range 0.11–1.0) and the median fibroblast SF_2_ was 0.49 (range 0.24–1.0). Comparing data from independent experiments, the overall SF_2_ was significantly correlated with the SF_2_ of fibroblasts (2*P* = 0.006) but not with the tumour cell SF_2_. The tumour cell and fibroblast radiosensitivities measured in the same individuals were not correlated (*r* = 0.06, 95% CI [–0.19, 0.30]). This finding seems to preclude a strong correlation between the radiosensitivity of tumour cells and fibroblasts. Concerning the clinical characteristics, neither of the measures of tumour radiosensitivity was correlated with T- and N-category, stage, tumour size, sex and age. However, the tumour cell radiosensitivity decreased with increasing grade of histopathological differentiation (2*P* = 0.012). The same tendency was found in two independent analyses of the same patient material. This correlation was not significant in case of the overall SF_2_ or the fibroblast SF_2_. © 1999 Cancer Research Campaign


*British Journal of Cancer* (1999) **79**(7/8), 1074–1084
© 1999 Cancer Research Campaign
Article no. bjoc.1998.0172

### In vitro radiosensitivity of tumour cells and fibroblasts derived from head and neck carcinomas: mutual relationship and correlation with clinical data

**B Stausbøl-Grøn[1], SM Bentzen[1], KE Jørgensen[2], OS Nielsen[3], T Bundgaard[4] and J Overgaard[1,3]**

[1]Danish Cancer Society, Department of Experimental Clinical Oncology, Aarhus University Hospital, Denmark; [2]Department of Ear, Nose and Throat Diseases, Odense University Hospital, Denmark; [3]Department of Oncology, Aarhus University Hospital, Denmark; [4]Department of Ear, Nose and Throat Diseases, Aarhus University Hospital, Denmark

**Summary** The aim was to characterize the variation in the cellular in vitro radiosensitivities in squamous cell carcinomas of the head and neck, and to test for a possible correlation between different measures of radiosensitivity and the clinical and histopathological data. Cellular in vitro radiosensitivities were assessed in tumour biopsies from 71 patients using the modified Courtenay–Mills soft agar clonogenic assay combined with an immunocytochemical analysis. Radiosensitivity was quantified as the surviving fraction after a radiation dose of 2 Gy irrespective of cell type (overall $SF_2$), or based on identification of cell type (tumour cell $SF_2$, fibroblast $SF_2$). Sixty-three biopsies were from primary tumours, and eight were from recurrences. Overall plating efficiency ranged from 0.005 to 1.60% with a median of 0.052%. The majority of the colonies obtained from the biopsies were fibroblast marker-positive; the proportion of tumour marker-positive colonies ranged from 1 to 88% with a median of 15%. The median overall $SF_2$ was 0.47 (range 0.24–0.96), the median tumour cell $SF_2$ was 0.50 (range 0.11–1.0) and the median fibroblast $SF_2$ was 0.49 (range 0.24–1.0). Comparing data from independent experiments, the overall $SF_2$ was significantly correlated with the $SF_2$ of fibroblasts ($2P = 0.006$) but not with the tumour cell $SF_2$. The tumour cell and fibroblast radiosensitivities measured in the same individuals were not correlated ($r = 0.06$, 95% CI [–0.19, 0.30]). This finding seems to preclude a strong correlation between the radiosensitivity of tumour cells and fibroblasts. Concerning the clinical characteristics, neither of the measures of tumour radiosensitivity was correlated with T- and N-category, stage, tumour size, sex and age. However, the tumour cell radiosensitivity decreased with increasing grade of histopathological differentiation ($2P = 0.012$). The same tendency was found in two independent analyses of the same patient material. This correlation was not significant in case of the overall $SF_2$ or the fibroblast $SF_2$.

**Keywords:** predictive assays; in vitro radiosensitivity; squamous cell carcinoma of the head and neck; differentiation; fibroblasts; tumour cells

Carcinoma of the head and neck is a loco-regional disease, which in many cases may be successfully treated by radiation. The outcome of curative radiotherapy depends on treatment parameters such as total dose, dose per fraction and overall treatment time. Furthermore, a number of tumour- and patient-related factors influence the outcome of radiotherapy, such as tumour site and size, stage and histology as well as patient age, sex and general condition. However, the outcome varies among patients to an extent that cannot be explained by these factors (Fertil and Malaise, 1981; Bentzen and Overgaard, 1994), and this observation motivates the search for new reliable predictors of treatment outcome. Such predictors could potentially allow individualization of radiotherapy and thereby, theoretically, improve the outcome both in the individual patient and at the patient population level.

One potential predictive factor for tumour outcome that has been studied is the clonogen surviving fraction of cells from tumour biopsies after a 2 Gy dose, the $SF_2$. In studies on both human tumour cell lines and primary cultures, $SF_2$ of different tumour histologies were ranked according to the clinical impression of radioresponsiveness (Fertil and Malaise, 1981; Deacon et al, 1984; Fertil and Malaise, 1985; Peters et al, 1988). This gave rise to studies searching for a correlation between different measures of tumour radiosensitivity and outcome after radiotherapy in individual patients. One study of carcinoma of the uterine cervix reported this kind of correlation using the modified Courtenay–Mills soft agar clonogenic assay (West et al, 1997). However, studies of head and neck carcinomas using the cell adhesion matrix (CAM) assay (Brock et al, 1989; Girinski et al, 1994; Eschwege et al, 1997) failed to demonstrate a correlation between tumour radiosensitivity and outcome after postoperative radiotherapy. Similarly, a lack of correlation was shown in studies using standard clonogenic assays in plastic dishes for early passage cell lines derived from glioblastoma multiforme, cervix and head and neck carcinomas (Allalunis-Turner et al, 1992; Schwartz et al, 1992; Taghian et al, 1992). Thus, it is still unclear whether tumour radiosensitivity, as it is measured with current assays, may predict the outcome of radiation treatment in individual patients.

Several studies have examined the relationship between in vitro radiosensitivity and tumour- and patient-related factors. Studies on carcinoma of the uterine cervix found measures of tumour radiosensitivity independent of tumour grade (Allalunis-Turner

*Received 11 November 1997*
*Revised 5 June 1998*
*Accepted 10 June 1998*

*Correspondence to:* B Stausbøl-Grøn, Danish Cancer Society, Department of Experimental Clinical Oncology, Aarhus University Hospital, Nørrebrogade 44, Building 5, DK-8000 Aarhus C, Denmark et al, 1991; West et al, 1997), tumour diameter, disease stage and patient age (West et al, 1997).

The current report is an extension of a study using the modified Courtenay–Mills soft agar clonogenic assay, to measure in vitro radiosensitivity in squamous cell carcinomas of the head and neck (SCCHN) (Stausbøl-Grøn et al, 1995). This previous study drew attention to the fact that stromal fibroblasts in addition to tumour cells were colony-forming in soft agar. An immunocytochemical analysis was developed to identify the origin of the colonies, and this provided estimates of the radiosensitivity of tumour cells (tumour cell $SF_2$), stromal fibroblasts (fibroblast $SF_2$), and an overall estimate (overall $SF_2$) in the same patient. Tumour cell $SF_2$ did not correlate with either overall $SF_2$ or fibroblast $SF_2$ (Stausbøl-Grøn et al, 1995).

In this paper, further data on the radiosensitivity of SCCHN are presented, and the relation to clinical and histopathological data is examined. In addition, the tumour cell and fibroblast radiosensitivities measured in the same tumour biopsies were compared.

### MATERIALS AND METHODS

### Patients and tumour biopsies

Tumour biopsies were obtained from 105 patients with SCCHN at the Department of Oncology, Aarhus University Hospital and the Ear, Nose and Throat Departments at Aarhus and Odense University Hospitals between March 1992 and November 1996. Informed consent according to the Helsinki Declaration II was obtained, and the study was approved by the local ethical committee. Several tumour biopsies were excluded from the analysis, and the reasons are listed in Figure 1.

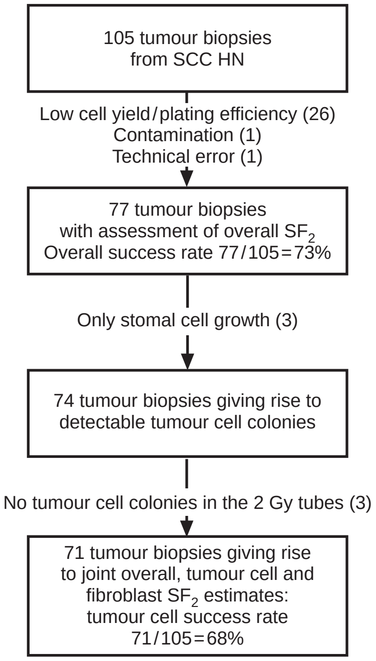

**Figure 1** The flow-chart shows the success rates and causes of failures for cultures of squamous cell carcinoma of the head and neck. The success rate for measuring tumour cell $SF_2$ was 68%. Low cell yield: fewer than two culture tubes could be set-up. Low plating efficiency: fewer than ten colonies in the unirradiated tubes

In tumour specimens from 71 patients, overall, $SF_2$ tumour cell and fibroblast $SF_2$ values (measured as joint estimates) were obtained. Sixty-three patients had primary tumours, and 38 patients were treated with curative radiotherapy alone. Eight patients had recurrent tumours, six after radiotherapy and two after surgery. Complete clinical data were available in 63 patients with primary disease. Among these patients, 18 were female. Median age was 61 years (range 26–91).

### Modified Courtenay–Mills soft agar clonogenic assay and immunocytochemical staining of colonies

Tumour samples for analysis were achieved under local or general anaesthesia. The presence of malignancy was checked by routine histological examination.

The cellular in vitro radiosensitivities were quantified by $SF_2$, that is the fraction of colony-forming cells after a dose of 2 Gy relative to that of an unirradiated sample. Tumour cell $SF_2$, fibroblast $SF_2$ and an overall $SF_2$ estimate were measured using a modified Courtenay–Mills soft agar clonogenic assay (Courtenay et al, 1978; Courtenay, 1984; West and Sutherland, 1986) and a colony-filter technique facilitating immunocytochemical staining, as previously described (Stausbøl-Grøn et al, 1995).

Tumour biopsies were disaggregated using collagenase, DNase, and pronase to form a single cell suspension. Following filtration and assessment of a viable cell count, the cells were plated in soft agar together with rat red blood cells and an enriched growth medium consisting of Hams F12, hydrocortisone, insulin, transferrin, epidermal growth factor and 15% fetal calf serum. The cells were irradiated 18–20 h after plating using a 250 kV Philips RT 305 X-ray apparatus yielding a dose rate of 1.59 Gy per min to the cells. After 4 weeks of incubation in a humidified atmosphere of 5% $O_2$, 5% $CO_2$, and 90% $N_2$, colonies > 60 μM in diameter were counted under a stereomicroscope.

After counting the colonies in each culture tube, the agar was strained off using a 30 μM filter and a Millipore filter system with vaccum filtration pressure. All the colonies in a culture tube were collected on a preparation slide. Immunocytochemical identification was obtained with different monoclonal antibodies on duplicate slides: broadspectrum anti-cytokeratin antibodies (AE 1–3 Cytokeratin, Biogenex) 1:40, reacting against the cytokeratins in human epithelia and carcinoma derived from these were used as a marker for tumour cells (Moll et al, 1982). Fibroblast colonies were identified using the 5B5 fibroblast antibody (Stausbøl-Grøn et al, 1995) or anti-vimentin antibody (3B4 1:200, M7020, DAKO). Anti-vimentin antibody reacts with vimentin, the intermediate filament protein present in cells of mesenchymal origin as, for example, fibroblasts (Azumi and Battifora, 1987). The validity of the monoclonal antibodies was tested on cytocentrifuge preparations and colony slides of an epithelial cell line and human fibroblast strains as described previously (Stausbøl-Grøn et al, 1995). Furthermore, tissue sections from SCCHN varying in tumour differentiation have been tested using both anti-cytokeratin and anti-vimentin antibodies. The method used for immunostaining was an avidin–biotin technique (Stausbøl-Grøn et al, 1995). After immunostaining, the colonies > 60 μM in diameter were counted and evaluated for staining reaction under a light microscope. As reported by Courtenay (1984), about 18 cells could be distinguished on the surface of a 50-cell colony seen from above or below using a × 20 objective to view the individual cells in a colony.

**Table 1** Tumour characteristics and parameters of in vitro radiosensitivity in 63 primary head and neck carcinomas

| Site | T | N classification | M | Stage | Size (mm) | Histopathological grade of differentiation | Plating efficiency (%) | Overall $SF_2$ | Tumour cell $SF_2$ | Fibroblast $SF_2$ |
|---|---|---|---|---|---|---|---|---|---|---|
| Oropharynx | 2 | 1 | 0 | 3 | 40 | Poor | 0.153 | 0.66 | 0.19 | 0.71 |
| Oropharynx | 3 | 3 | 1 | 4 | 60 | Poor | 0.039 | 0.65 | 0.33 | 0.55 |
| Oropharynx | 2 | 1 | 0 | 3 | 30 | Moderate | 0.021 | 0.58 | 0.25 | 0.50 |
| Oral cavity | 4 | 2 | 0 | 4 | 40 | Poor | 0.214 | 0.35 | 0.11 | 0.39 |
| Oral cavity | 3 | 0 | 0 | 3 | 50 | Poor | 0.046 | 0.40 | 0.30 | 0.40 |
| Oropharynx | 2 | 0 | 0 | 2 | 25 | Poor | 0.036 | 0.24 | 0.67 | 0.26 |
| Oral cavity | 2 | 0 | 0 | 2 | 20 | Moderate | 0.181 | 0.46 | 0.28 | 0.47 |
| Oropharynx | 1 | 0 | 0 | 1 | 10 | Moderate | 0.019 | 0.44 | 0.25 | 0.46 |
| Oropharynx | 2 | 0 | 0 | 2 | 25 | Poor | 0.074 | 0.45 | 0.50 | 0.53 |
| Oral cavity | 2 | 0 | 0 | 2 | 30 | Well | 0.313 | 0.41 | 0.24 | 0.48 |
| Oropharynx | 2 | 2 | 0 | 4 | 30 | Poor | 0.022 | 0.77 | 0.50 | 0.77 |
| Oral cavity | 1 | 0 | 0 | 1 | 15 | Moderate | 0.040 | 0.45 | 0.36 | 0.46 |
| Hypopharynx | 4 | 0 | 0 | 4 | 40 | Moderate | 0.168 | 0.46 | 0.36 | 0.44 |
| Oral cavity | 2 | 0 | 0 | 2 | 25 | Moderate | 0.108 | 0.70 | 0.38 | 0.69 |
| Oropharynx | 4 | 0 | 0 | 4 | 70 | Moderate | 0.050 | 0.43 | 0.41 | 0.65 |
| Oral cavity | 1 | 0 | 0 | 1 | 18 | Well | 0.053 | 0.56 | 0.75 | 0.60 |
| Supraglottic | 4 | 0 | 0 | 4 | 35 | Poor | 0.023 | 0.42 | 0.41 | 0.41 |
| Oropharynx | 1 | 0 | 0 | 1 | 15 | Poor | 1.447 | 0.48 | 0.46 | 0.49 |
| Oral cavity | 3 | 1 | 0 | 3 | 60 | Well | 0.005 | 0.61 | 0.50 | 0.75 |
| Oral cavity | 2 | 0 | 0 | 2 | 40 | Well | 0.013 | 0.57 | 0.50 | 0.58 |
| Hypopharynx | 2 | 0 | 0 | 2 | 40 | Moderate | 0.021 | 0.68 | 0.50 | 0.44 |
| Oropharynx | 2 | 2 | 1 | 4 | 10 | Poor | 0.017 | 0.77 | 0.67 | 0.66 |
| Oropharynx | 3 | 1 | 0 | 3 | 45 | Poor | 0.026 | 0.67 | 0.56 | 0.65 |
| Oral cavity | 2 | 0 | 0 | 2 | 30 | Moderate | 0.027 | 0.75 | 0.43 | 0.77 |
| Oropharynx | 2 | 0 | 0 | 2 | 30 | Moderate | 0.037 | 0.56 | 0.67 | 0.57 |
| Oropharynx | 3 | 0 | 0 | 3 | 50 | Poor | 0.016 | 0.57 | 0.48 | 1.00 |
| Oral cavity | 1 | 0 | 0 | 1 | 15 | Well | 1.596 | 0.35 | 0.53 | 0.38 |
| Hypopharynx | 2 | 2 | 0 | 4 | 30 | Poor | 0.291 | 0.40 | 0.56 | 0.49 |
| Oral cavity | 1 | 0 | 0 | 1 | 20 | Well | 0.200 | 0.41 | 0.49 | 0.37 |
| Oral cavity | 2 | 0 | 0 | 2 | 30 | Well | 0.014 | 0.73 | 0.75 | 0.78 |
| Oral cavity | 1 | 0 | 0 | 1 | 15 | Well | 0.219 | 0.46 | 0.43 | 0.49 |
| Oropharynx | 2 | 0 | 0 | 2 | 25 | Poor | 0.015 | 0.75 | 0.59 | 0.81 |
| Oropharynx | 2 | 2 | 0 | 4 | 30 | Moderate | 0.027 | 0.42 | 0.59 | 0.43 |
| Oral cavity | 1 | 0 | 0 | 1 | 20 | Well | 0.214 | 0.39 | 0.54 | 0.32 |
| Oral cavity | 2 | 0 | 0 | 2 | 35 | Well | 0.149 | 0.32 | 0.65 | 0.45 |
| Oropharynx | 1 | 0 | 0 | 1 | 20 | Well | 0.031 | 0.52 | 0.58 | 0.62 |
| Oropharynx | 3 | 1 | 0 | 3 | 50 | Moderate | 0.014 | 0.60 | 0.46 | 0.63 |
| Oral cavity | 1 | 0 | 0 | 1 | 10 | Moderate | 0.290 | 0.51 | 0.66 | 0.47 |
| Oral cavity | 4 | 2 | 0 | 4 | 75 | Poor | 0.143 | 0.49 | 0.69 | 0.50 |
| Oral cavity | 2 | 0 | 0 | 2 | 35 | Moderate | 0.178 | 0.42 | 0.64 | 0.26 |
| Oropharynx | 2 | 0 | 0 | 2 | 40 | Well | 0.018 | 0.94 | 1.00 | 0.90 |
| Glottic | 2 | 0 | 0 | 2 | 10 | Moderate | 0.044 | 0.96 | 0.93 | 0.80 |
| Glottic | 1 | 0 | 0 | 1 | 5 | Moderate | 0.117 | 0.33 | 0.75 | 0.41 |
| Oral cavity | 4 | 3 | 0 | 4 | 30 | Poor | 0.094 | 0.60 | 0.78 | 0.57 |
| Supraglottic | 3 | 2 | 0 | 4 | 40 | Well | 0.041 | 0.76 | 1.00 | 0.67 |
| Oral cavity | 2 | 0 | 0 | 2 | 30 | Well | 0.042 | 0.51 | 0.83 | 0.54 |
| Oral cavity | 1 | 0 | 0 | 1 | 12 | Well | 0.141 | 0.67 | 0.87 | 0.44 |
| Supraglottic | 3 | 0 | 0 | 3 | 50 | Well | 0.005 | 0.79 | 1.00 | 0.72 |
| Oropharynx | 1 | 0 | 0 | 1 | 20 | Moderate | 0.253 | 0.39 | 1.00 | 0.39 |
| Oral cavity | 2 | 1 | 0 | 3 | 30 | Moderate | 0.033 | 0.37 | 0.50 | 0.34 |
| Maxillary sinus | 4 | 0 | 0 | 4 | 60 | Well | 0.029 | 0.46 | 0.72 | 0.50 |
| Oral cavity | 2 | 0 | 0 | 2 | 30 | Well | 0.095 | 0.30 | 0.50 | 0.25 |
| Oral cavity | 3 | 1 | 0 | 3 | 50 | Well | 0.058 | 0.49 | 1.00 | 0.46 |
| Oropharynx | 3 | 2 | 0 | 4 | 45 | Poor | 1.026 | 0.38 | 0.23 | 0.39 |
| Oral cavity | 1 | 0 | 0 | 1 | 20 | Moderate | 0.024 | 0.48 | 0.30 | 0.45 |
| Oral cavity | 1 | 0 | 0 | 1 | 20 | Well | 0.046 | 0.38 | 0.44 | 0.35 |
| Oropharynx | 1 | 2 | 0 | 4 | 20 | Poor | 0.650 | 0.36 | 0.34 | 0.36 |
| Oral cavity | 1 | 0 | 0 | 1 | 20 | Well | 0.118 | 0.38 | 0.31 | 0.38 |
| Oral cavity | 2 | 0 | 0 | 2 | 30 | Well | 0.125 | 0.52 | 0.52 | 0.52 |
| Oral cavity | 2 | 0 | 0 | 2 | 25 | Well | 0.038 | 0.47 | 0.50 | 0.49 |
| Oropharynx | 1 | 0 | 0 | 1 | 15 | Moderate | 0.293 | 0.51 | 0.15 | 0.55 |
| Oropharynx | 1 | 0 | 0 | 1 | 20 | Well | 0.788 | 0.27 | 0.75 | 0.24 |
| Oral cavity | 4 | 0 | 0 | 4 | 30 | Well | 0.621 | 0.38 | 0.57 | 0.36 |

*© Cancer Research Campaign 1999*

### Statistical analysis and calculation of the surviving fraction after 2 Gy

Colony counts were assumed to have a Poisson distribution, and the standard errors of ratios (like $SF_2$) were calculated using the normal distribution approximation.

Generally, both the data from the cytokeratin and vimentin experiments were used to estimate the surviving fractions in order to reduce the statistical uncertainty of the radiosensitivity estimate of tumour cells and fibroblasts (Stausbøl-Grøn et al, 1995). However, the calculation of the surviving fractions was modified compared to the previous paper (Stausbøl-Grøn et al, 1995), since the larger sample in the current work made it possible to compare the two staining procedures statistically. Instead of adding the colony counts from the two procedures, two separate $SF_2$ values were estimated, from which a mean value weighted by their inverse variances was calculated to make a joint estimate. A motivation for this procedure is given in the Result section below.

The fibroblast colonies were defined as the colonies that were positively stained with anti-vimentin antibody or were negatively stained with the anti-cytokeratin antibody. In the same way, the epithelial tumour colonies were defined as cytokeratin-positive colonies or vimentin-negative colonies. If no colonies were obtained in the 2 Gy tubes from the two staining procedures, the radiosensitivity in question was not achieved. However, if only one of the $SF_2$ measures was defined, this measure was accepted as the joint measure of radiosensitivity.

Linear regression was used to test the relationship between continuous variables of the surviving fraction of tumour cells, fibroblasts and overall estimates independently measured. Tests involving tumour cell $SF_2$ were done by weighted linear regression, weighted with the inverse variance of the tumour cell $SF_2$. Thereby, the low number of tumour cell colonies was taken into account by weighting the best-determined tumour cell $SF_2$ values the most. When correlating the radiosensitivities independently measured in the individual patients, the tumour cell $SF_2$ was calculated from the positive colonies in the cytokeratin-stained slides or from the negative colonies in the vimentin-stained slides. Similarly, the fibroblast $SF_2$ was calculated from the positive colonies in the vimentin-stained slides or from the negative colonies in the cytokeratin-stained slides. The overall $SF_2$ estimates were calculated from the total number of colonies in the cytokeratin- or in the vimentin-stained slides.

Spearman correlation test was used to examine the relationship between the in vitro radiosensitivities and ordinal clinical and histopathological parameters, e.g. stage or grade. In all cases, a significance level of 5% was used. All *P*-values given in the text are from two-sided tests.

### Clinical and histopathological parameters

In the analyses, the site of the primary tumours was classified according to the International Union against Cancer's (UICC) TNM (Tumour, Node, Metastasis) classification of malignant tumours (1987). Similarly for staging, which was obtained by clinical examination, X-ray examination and computed tomography. Tumour size was recorded as the largest dimension measured clinically.

For routine histopathological examination, specimens were obtained mostly in parallel with the specimen for radiosensitivity testing. Histological sections (3 µM) were cut from formalin-fixed and paraffin-embedded specimens, and histopathological differentiation was graded as well, moderately and poorly differentiated, according to Broders (1920).

The clinical and histopathological characteristics in addition to the radiosensitivity data in 63 patients with primary tumours are listed in Table 1.

### RESULTS

### Validation of the assay for radiosensitivity testing

In addition to the modified Courtenay–Mills soft agar clonogenic assay, an immunocytochemical analysis was used and evaluated in the current study.

Biopsy material was obtained from 105 patients with SCCHN. In 26 cases, the cell yield was too low for plating two culture tubes, or plating efficiency was low with fewer than ten colonies in the unirradiated tubes. Data on the overall in vitro radiosensitivity was obtained in 77 patient biopsies, which all met the criteria for successful growth with more than ten colonies in the unirradiated tubes. The overall success rate was 73%. However, only stromal fibroblast growth was obtained in three of the tumour biopsies. Hence, it was possible to detect tumour cell growth in 74 tumour biopsies. But, in three of the tumour biopsies, no tumour cell colonies were obtained in the 2-Gy tubes and these were excluded from analyses of $SF_2$ values (Figure 1). The plating efficiency for all cells (e.g. tumour cells and fibroblasts) ranged from 0.005 to 1.60% with a median of 0.052%.

The reliability of the immunocytochemical analysis using cytokeratin and vimentin antibodies to detect epithelial cells and fibroblasts, respectively, was tested on sections of SCCHN (Figure 2). Using the colony-filter technique and immunocytochemistry, it was possible to estimate the numbers of both tumour cell colonies and fibroblast colonies. Typical tumour cell colonies and fibroblast colonies are shown in Figure 2. The majority of the colonies obtained from the tumour biopsies were fibroblast marker-positive, and the tumour marker-positive colonies ranged from 1 to 88% with a median of only 15% in the control tubes (Figure 3).

Due to the larger number of patients compared to the previous paper (Stausbøl-Grøn et al, 1995), the methodological quality of the immunocytochemical staining using two monoclonal antibodies could be evaluated statistically. Before estimating a joint $SF_2$ value from the results of the two staining procedures, the monoclonal antibodies ought to provide the same measure of colony-forming capacity both in the control and the irradiated tubes. As shown in Figure 4, colony counts from the two staining procedures were significantly correlated and proportional, but for the fibroblast colonies, the slope of the regression line was significantly different from 1. This may suggest an observer bias where negative colonies were overlooked to some extent. However, the $SF_2$ value is the ratio between colony counts at 2 Gy and 0 Gy. Therefore, the constant of proportionality cancels out, and the $SF_2$ values estimated from the two staining procedures should be comparable. Thus, both measures of radiosensitivity were entered in the calculations, and a mean value weighted by the separate inverse variances was calculated to take into account the uncertainty in the separate $SF_2$ measures.

The joint estimates of radiosensitivity varied considerably. The overall $SF_2$ ranged from 0.24 to 0.96 with the median overall $SF_2$ being 0.47, the median tumour cell $SF_2$ was 0.50 (range 0.11–1.0) and the median fibroblast $SF_2$ was 0.49 (range 0.24–1.0).

© Cancer Research Campaign 1999

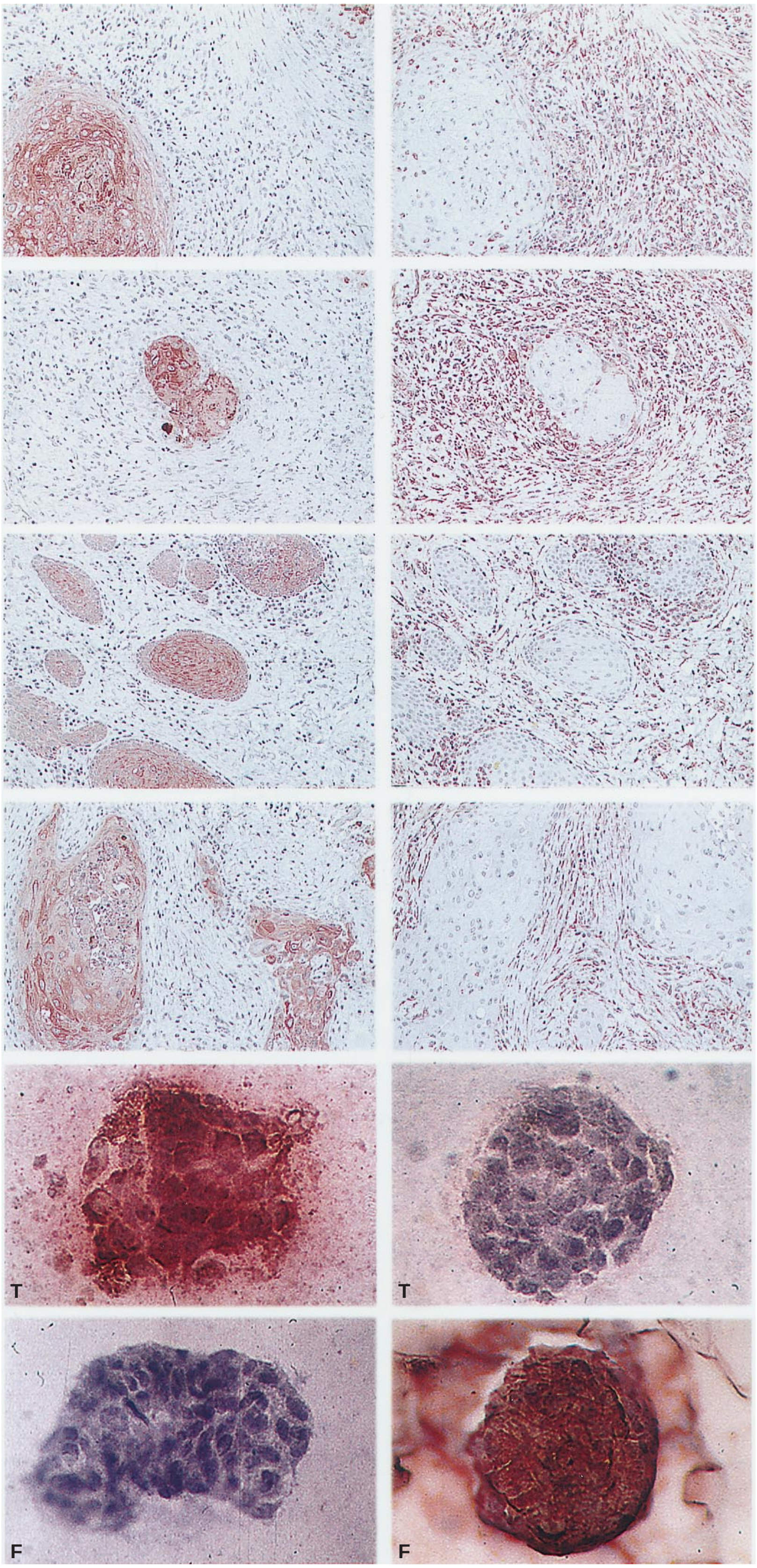

**Figure 2** Immunostaining of serial sections (× 50) and colonies (× 400) obtained from pretreatment head and neck squamous cell carcinoma specimens. Left panel: expression of cytokeratin using cytokeratin antibodies AE1-3 (1:40). Right panel: vimentin expression using vimentin antibody 3B4 (1:200). The colonies are typical tumour cell colonies (T) and fibroblast colonies (F), respectively, according to the definitions in the text

*© Cancer Research Campaign 1999*

It was not possible to examine for intra-tumour heterogeneity, since the tumour material obtained was limited. However, the relationship between independent measures of $SF_2$ obtained within the same tumour biopsy was examined using a linear regression analysis (Table 2). Sufficient colony tubes/slides for staining with both anti-cytokeratin and anti-vimentin were obtained in 71 patients. The assay reproducibility was tested by comparing one measure of overall $SF_2$ (total number of colonies in anti-cytokeratin-stained slides) with another measure of overall $SF_2$ (total number of colonies in anti-vimentin-stained slides), and these measures were significantly correlated. The fibroblast $SF_2$ (fibroblast marker-positive colonies) and a fibroblast $SF_2$ measured independently of the latter (tumour marker-negative colonies) correlated significantly in 69 patients. Furthermore, in 43 patients with two independent measures of tumour cell $SF_2$, the variation in tumour cell $SF_2$ was examined using weighted linear regression, to be able to give the best determined $SF_2$ value the highest weight. Tumour cell $SF_2$ (tumour marker-positive colonies) was significantly correlated to another measure of tumour cell $SF_2$ (fibroblast marker-negative colonies).

In conclusion, the present assay for radiosensitivity testing was validated, and it was reproducible for overall $SF_2$, fibroblast $SF_2$

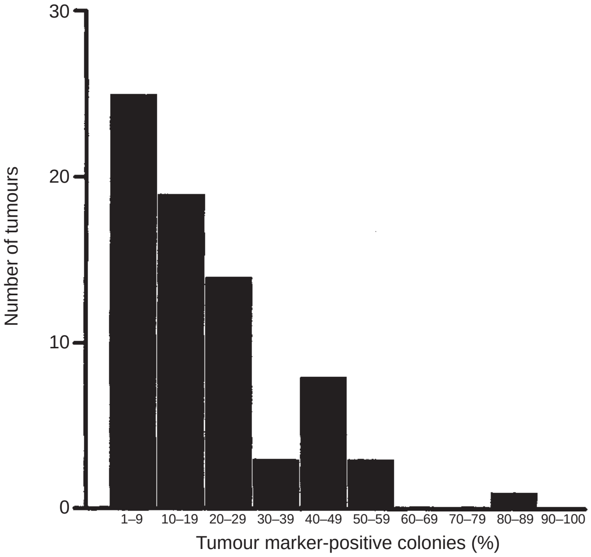

**Figure 3** The percentage of tumour cell colonies out of all colonies in the unirradiated tubes. One patient biopsy was omitted from the figure, since no positive colonies were obtained in the tumour marker-stained slides; $n = 73$

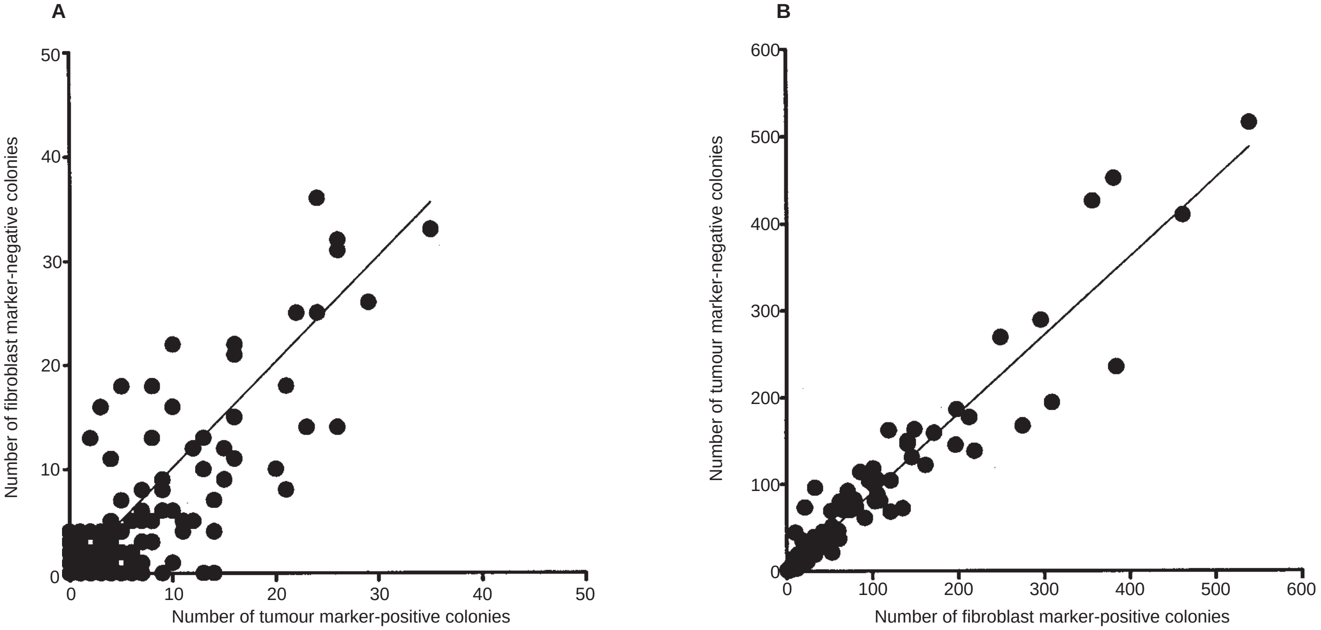

**Figure 4** The correlation between the number of colonies in control and 2-Gy tubes in 71 biopsies stained with both anti-cytokeratin and anti-vimentin: (**A**) Tumour cell colonies: $n = 142$, $r = 0.70$, $2P < 0.001$, the slope of the regression line = 1.02. The points (14.83) and (31.71) are not shown. Slope when leaving out these points = 0.85. (**B**) Fibroblast colonies: $n = 142$, $r = 0.96$, $2P < 0.001$, the slope of the regression line = 0.91, $2P < 0.001$

*© Cancer Research Campaign 1999*

**Table 2** Correlation between independent experiments in the same tumour biopsy examining reproducibility

| Variables | | *n* | *r* | 2*P*-value | Analysis |
|---|---|---|---|---|---|
| Overall $SF_2$ (ck) | Overall $SF_2$ (vim) | 71 | 0.55 | < 0.001 | Linear regression |
| Fibroblast $SF_2$ (vim+) | Fibroblast $SF_2$ (ck–) | 69[a] | 0.43 | 0.001 | Linear regression |
| Tumour cell $SF_2$ (ck+) | Tumour cell $SF_2$ (vim–) | 43[b] | 0.31 | 0.046 | Weighted regression[c] |

[a]Fibroblast $SF_2$ (ck–) with non-zero colony count at 2 Gy. [b]Tumour cell $SF_2$ (ck+) and tumour cell $SF_2$ (vim–) with non-zero colony count at 2 Gy. [c]Weighted with the statistical precision on tumour cell $SF_2$ (ck+) and tumour cell $SF_2$ (vim–). ck = tumour marker; vim = fibroblast marker; +/– = positive/negative reaction

**Table 3** Correlation between measures of radiosensitivity with respect to cell type in the same individual

| Variables | | *n* | *r* | 2*P*-value | Analysis |
|---|---|---|---|---|---|
| Overall $SF_2$ (ck) | Fibroblast $SF_2$ (vim+) | 71 | 0.33 | 0.006 | Linear regression |
| Overall $SF_2$ (vim) | Tumour cell $SF_2$ (ck+) | 63[a] | 0.11 | 0.388 | Weighted regression[b] |
| Fibroblast $SF_2$ (vim+) | Tumour cell $SF_2$ (ck+) | 63[a] | 0.06 | 0.643 | Weighted regression[b] |

[a]Tumour cell $SF_2$ (ck+) with non-zero colony count at 2 Gy. [b]Weighted with the statistical precision on tumour cell $SF_2$ (ck+). ck = tumour marker; vim = fibroblast marker; + = positive reaction.

and tumour cell $SF_2$. Among patients, it was possible to measure a large variation in inter-tumour in vitro radiosensitivity of tumour cells and fibroblasts, and the overall estimate. Finally, the main problems displayed were the low plating efficiencies and the low number of tumour cell colonies, when growing cells from tumour specimens in the modified Courtenay–Mills soft agar clonogenic assay.

### Relationship between the radiosensitivity of tumour cells and fibroblasts

This study was an attempt to investigate the radiation biology in tumours, and to compare the in vitro radiosensitivity of fibroblasts and tumour cells.

In order to ensure statistical independence of the variables, a linear regression analysis was performed to test the relationship between various measures of $SF_2$. Table 3 shows a comparison of the separately measured radiosensitivities of tumour cells and fibroblasts, and an overall estimate. In 71 patients, overall $SF_2$ (total number of colonies in anti-cytokeratin-stained slides) correlated significantly with the fibroblast $SF_2$ (fibroblast marker-positive colonies) (Figure 5A). This meant that the standard measure of tumour radiosensitivity in many studies, the overall $SF_2$, was correlated with the sensitivity of the stromal fibroblasts. In contrast, the overall $SF_2$ (total number of colonies in anti-vimentin-stained slides) did not correlate with the tumour cell $SF_2$ (tumour marker-positive colonies) in 63 patients using weighted linear regression, taking into account the statistical precision of tumour cell $SF_2$ (Figure 5B).

Tumour cell $SF_2$ (tumour marker-positive colonies), and fibroblast $SF_2$ (fibroblast marker-positive colonies) measured in the same individual were not correlated. The correlation coefficient was 0.06 with 95% confidence interval (CI) (–0.19; 0.30) (Figure 5C). A previous published paper on 12 of the patients also demonstrated a lack of correlation between the radiosensitivity of tumour cells and fibroblasts (Stausbøl-Grøn et al, 1995) and this was confirmed in an analysis leaving out the first 12 patients ($r$ = 0.06, 95% CI [–0.21; 0.33]). Thus, the data preclude any strong correlation between tumour and fibroblast radiosensitivity as assessed here.

### Relationship between cellular in vitro radiosensitivities and clinical data

Table 1 shows the tumour characteristics and the parameters of radiosensitivity in SCCHN from 63 patients with primary disease. Large tumours were found to have lower overall plating efficiencies than small tumours ($n$ = 63, $r$ = –0.32, 2$P$ = 0.011). The same correlation was found for T-category when related to plating efficiency ($n$ = 63, $r$ = –0.28, 2$P$ = 0.027). Furthermore, plating efficiencies were higher in tumours from younger patients than older patients ($n$ = 63, $r$ = –0.32, 2$P$ = 0.011). No correlation was found between overall plating efficiency and overall $SF_2$, tumour cell $SF_2$ or fibroblast $SF_2$.

Overall $SF_2$ and tumour cell $SF_2$ were not correlated with T- and N-category, stage, tumour size, sex and age. However, the tumour cell $SF_2$ values were lower in the poorly differentiated tumours than in the well- and moderately differentiated tumours, which suggests that the well-differentiated tumours were more radioresistant (Figure 6). This relationship was not statistically significant for the overall $SF_2$ (2$P$ = 0.65), or the fibroblast $SF_2$ (2$P$ = 0.38). Obviously, this finding should be seen in light of the rather high number of statistical tests performed. However, tumour cell radiosensitivity increased with decreasing tumour differentiation ($r$ = –0.34, 2$P$ = 0.056) in our previous study on 31 patients with SCCHN (Stausbøl-Grøn et al, 1996). Excluding the 31 patients from the analysis of the 63 patients with primary tumours in the current study, the same trend was found in the remaining patients ($r$ = –0.34,

 *© Cancer Research Campaign 1999*

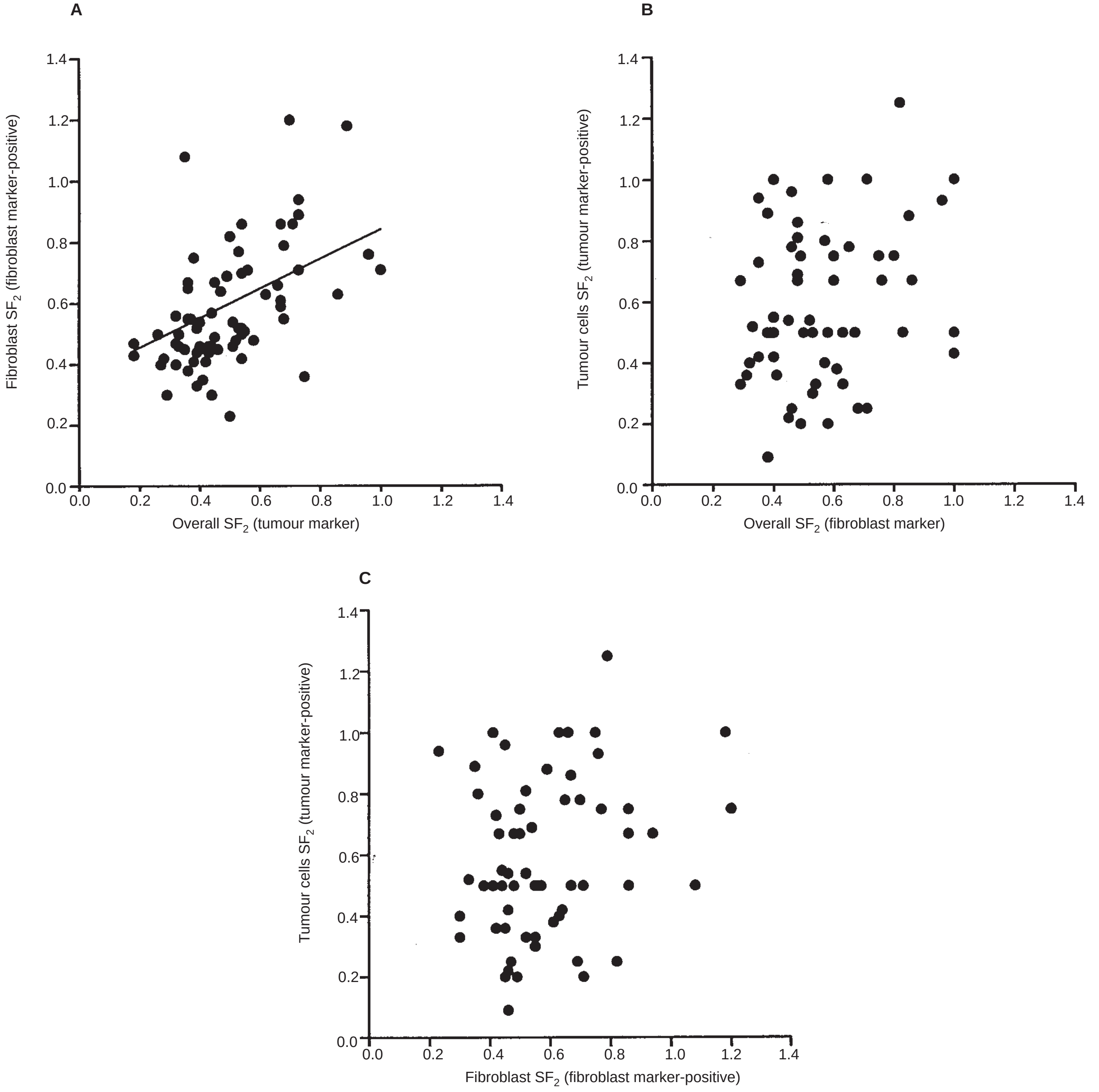

**Figure 5** The relationship between different measures of in vitro radiosensitivity in the same individual with respect to cell-type. (**A**) A correlation between overall $SF_2$ and fibroblast $SF_2$ was found ($n = 71$, $r = 0.33$, $2P = 0.006$; (0.33, 2.00), not shown). (**B**) Overall $SF_2$ and tumour cell $SF_2$ did not correlate ($n = 63$, $r = 0.11$, $2P = 0.39$). (**C**) No correlation was found between the tumour cell radiosensitivity and the fibroblast radiosensitivity ($n = 63$, $r = 0.06$, $2P = 0.64$; (2.00, 0.43), not shown)

$2P = 0.052$). Thus, the consistency of two independent analyses adds credibility to the finding.

### DISCUSSION

In vitro radiosensitivity of tumour cells and fibroblasts within an individual were not correlated in squamous cell carcinomas of the head and neck under the assay conditions described above. In addition, the poorly differentiated tumours were more radiosensitive, measured by tumour cell $SF_2$, than the well- and moderately differentiated tumours. To judge the representativeness of these findings, it is of interest to compare our assay with reports in the literature.

The assay used in the current study was similar to the often used modified Courtenay–Mills soft agar clonogenic assay (West et al, 1989, 1993), except for the addition of an immunocytochemical analysis for staining colonies from soft agar (Stausbøl-Grøn et al, 1995). The 73% overall success rate in this study is consistent with the overall success rate in previous studies on cellular in vitro radiosensitivity of different tumour types using either CAM assays

*© Cancer Research Campaign 1999*

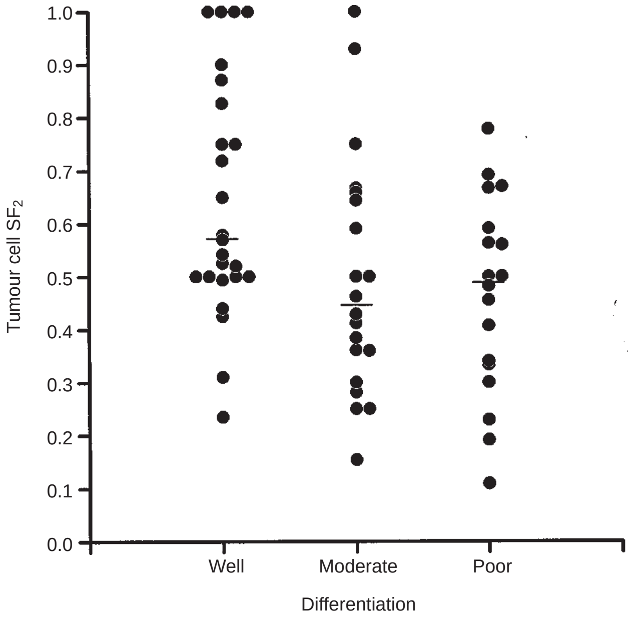

**Figure 6** The correlation between tumour cell radiosensitivity and the histopathological grade of differentiation in 63 primary tumours (*r* = –0.32, 2*P* = 0.012); bars = median or soft agar clonogenic assays (Brock et al, 1990; Girinski and Fertil, 1993; Kocagöncü et al, 1994; West, 1995). The success rate for measuring tumour cell radiosensitivity was 68%. Furthermore, the plating efficiency and the overall $SF_2$ values measured in this study fell within the same range as in similar studies on in vitro radiosensitivity (Brock et al, 1989; West et al, 1989, 1993; Girinski et al, 1994; Kocagöncü et al, 1994; West, 1995).

Measuring cell-type specific radiosensitivity required the use of monoclonal antibodies. Cytokeratins are characteristic of epithelial cells (Moll et al, 1982), and anti-cytokeratin antibodies with broad specificity were used as tumour cell markers, whereas 5B5 fibroblast antibody and anti-vimentin antibody were used as fibroblast markers. Presumably, it may be an advantage to use a panel of antibodies to different relevant markers, e.g. different intermediate filaments (Suo et al, 1992) for identifying cell types with a reasonable degree of certainty. Haustermans et al (1996) have identified cytokeratin antibodies that will stain a high proportion of head and neck cancers with no, or very little, staining of stromal cells. However, the ability of tumours to show coexpression of cytokeratin and vimentin has been demonstrated in some carcinomas (Azumi and Battifora, 1987). This may constitute a pitfall and influence the interpretation of the results. In this study, immunostaining of sections from SCCHN showed reliable staining. The anti-cytokeratin antibodies stained epithelia and tumour cells, and anti-vimentin antibody stained stromal cells. Such a reasonable agreement between the reactions of these antibodies is a prerequisite for using the data from both markers.

In contrast to Davidson et al (1990), it was not possible to examine tumour heterogeneity due to limited tumour material. Yet, within each single tumour biopsy separately measured overall $SF_2$ (fibroblast marker/tumour marker), fibroblast $SF_2$ (fibroblast marker-positive/tumour marker-negative colonies) and tumour cell $SF_2$ (tumour marker-positive/fibroblast marker-negative colonies) estimates correlated significantly. But the low correlation coefficients suggest a high level of noise in the modified Courtenay–Mills soft agar clonogenic assay. Furthermore, the immunocytochemical analysis indicated that only a small fraction of the colonies was tumour marker-positive when culturing cells from SCCHN. Approximately 70% of the tumours yielded less than 20% tumour cell colonies in both the control and the irradiated tubes. This low number of tumour cell colonies causes a considerable uncertainty in the measure of tumour cell radiosensitivity estimating tumour cells $SF_2$ values of 1 and more, due to random fluctuations in the colony counts at 0 and 2 Gy. One may exclude the tumour biopsies that reach less than a certain number of tumour cell colonies from the analysis, but the cut-off point will be arbitrary. An alternative may be to take all the available data into account and calculate a mean $SF_2$ value weighted by the inverse variances, and to do weighted linear regression tests weighted with the statistical precision on $SF_2$. In this study, the weighted linear regression test was preferred in the case of the tumour cell $SF_2$, because of the low number of tumour cell colonies. This meant that the best determined $SF_2$ values were given more weight. However, a minimum requirement of three tumour cell colonies in the culture tubes was implemented. This resulted in the exclusion of many patients, but the main conclusions were unchanged. Finally, it may be concluded that the low number of tumour cell colonies and low plating efficiencies are the main methodological problems when culturing primary specimens from SCCHN in the modified Courtenay–Mills soft agar clonogenic assay.

The current method has been shown to be suitable for studies of fibroblast and tumour cell radiosensitivity in the same tumour and individual, which in turn may provide valuable biological insights into the possible correlation between the sensitivity of normal and tumour cells (Stausbøl-Grøn et al, 1998). Individual in vitro radiosensitivities of tumour cells and fibroblasts as assessed here were not correlated in SCCHN. In agreement with this finding, in vitro studies reviewed by Bentzen and Overgaard (1994) found a lack of correlation between the in vitro radiosensitivity of various normal cell types in the same person. However, one study has reported a link between the in vitro radiosensitivity of fibroblasts and sarcoma cells in the same patient (Dahlberg et al, 1993).

A range of clinical studies (Tucker et al, 1992; Bentzen and Overgaard, 1994; Geara et al, 1996; Bentzen, 1997; Bernier et al, 1998) have suggested that the individual differences in radiosensitivity are not dominated by a common genetically determined factor expressed equally in all cells. This provides indirect support for a real biological difference in tumour cell and fibroblast radiosensitivity in SCCHN. Two studies did find a correlation between individual patient sensitivity of early mucosal reactions and radiation response of tumours derived from the same tissue (Bujko et al, 1994; Dahl et al, 1994), but these findings may need to be validated by other studies (Bentzen, 1997). Thus, a correction for the contamination of fibroblasts and a separate measurement of tumour cell radiosensitivity may be necessary, when studying tumour radiosensitivity and tumour response to radiation, but to what extent tumour cell $SF_2$ is predictive for treatment outcome is still not clear.

If the cellular in vitro radiosensitivity is to be a clinically useful predictive parameter, it must supplement the information from known prognostic factors. Yet, a lack of correlation between in vitro radiosensitivity and clinical data may be influenced by fibroblast contamination in the cultures. In this study, no significant correlations were found between overall $SF_2$ and clinical and

© Cancer Research Campaign 1999

histopathological parameters. Furthermore, tumour cell radiosensitivity and T- and N-category, stage, tumour size, sex and age were not significantly associated.

However, in vitro radiosensitivity of tumour cells and tumour differentiation were statistically significantly correlated with the well- and moderately differentiated tumours being more radioresistant than the poorly differentiated tumours. But the correlation was not conspicuous, as seen from Figure 4. One may argue whether this relationship is a biological finding, suggesting that the tumour cell $SF_2$ is a more reliable measure of tumour radiosensitivity than the overall $SF_2$, or simply a result of multiple comparisons. In analysis of many parameters, the probability of finding a significant difference just by chance increases with the number of tests performed (Beck-Bornholdt and Dubben, 1994). However, in this study a subset consisting of 31 patients was previously analysed in what might be regarded as a hypothesis-generating analysis (Stausbøl-Grøn et al, 1996). A relationship between tumour cell $SF_2$ and differentiation was seen with a tendency for well-differentiated tumours to be more resistant. This trend was also seen in an independent group of the remaining patients, which supports that this is not just a spurious finding.

Recently, it was suggested that well-differentiated carcinomas of the head and neck respond to a radiation trauma with accelerated repopulation just like the normal mucosa, and that these tumours may benefit from reduced overall treatment time (Hansen et al, 1997). In the present study, the poorly differentiated tumours appeared to be the most radiosensitive, measured by tumour cell $SF_2$. Taken together, these observations, if substantiated, could argue in favour of a reduced overall treatment time, even at the expense of a decreased total dose such as the randomized trial of continuous, hyperfractionated, accelerated radiotherapy (CHART) (Dische et al, 1997).

Finally, it remains to be tested whether estimates of radiosensitivity are useful predictors of the response to radiotherapy. Unfortunately, it may not be possible to test if measured fibroblast radiosensitivity predicts normal tissue response in patients, since the clinical data have not been collected. The low number of tumour cell colonies obtained from primary tumours in the modified Courtenay–Mills soft agar clonogenic assay may result in a too uncertain measure of tumour cell radiosensitivity to allow prediction of the tumour response to radiotherapy. However, it is well-established that the tumour response is dependent on other factors, for instance tumour oxygenation status (Nordsmark et al, 1996) and patient-related factors (Bentzen and Overgaard, 1994), probably making it difficult to predict clinical outcome from cellular in vitro radiosensitivity alone.

### ACKNOWLEDGEMENTS

The authors would like to thank Dr Helmer Søgaard at the Department of Pathology, Aarhus University Hospital, for excellent assistance in assessment of the histopathological grading, Ms Alice Baden for valuable technical assistance, and Dr Olfred Hansen at the Department of Oncology, Odense University Hospital, for assistance in obtaining some of the clinical data. Finally, thanks to Dr Michael R Horsman for critically reading the manuscript. This work was supported by grants from the Danish Cancer Society, The Clinical Research Unit at the Oncology Center, Aarhus University Hospital and The Faculty of Health Sciences, Aarhus University, Denmark.

### REFERENCES

Allalunis-Turner M, Pearcey RG, Barron GM, Buryn DA, Babiak JC and Honoré LH (1991) Inherent radiosensitivity testing of tumor biopsies obtained from patients with carcinoma of the cervix or endometrium. *Radiother Oncol* **22**: 201–205

Allalunis-Turner M, Day R, Pearcey R and Urtasun R (1992) Radiosensitivity testing in gynecological tumours and malignant gliomas. In *Radiation Research: A Twentieth Century Perspective*, Dewey W, Edington M, Fry R, Hall E and Whitmore G (eds), p. 712–715. Academic Press: San Diego

Azumi N and Battifora H (1987) The distribution of vimentin and keratin in epithelial and nonepithelial neoplasms. *Am J Clin Pathol* **88**: 286–296

Beck-Bornholdt HP and Dubben HH (1994) Potential pitfalls in the use of *P*-values and in interpretation of significance levels. *Radiother Oncol* **33**: 171–176

Bentzen SM (1997) Potential clinical impact of normal-tissue intrinsic radiosensitivity testing. *Radiother Oncol* **43**: 121–131

Bentzen SM and Overgaard J (1994) Patient-to-patient variability in the expression of radiation-induced normal tissue injury. *Semin Radiat Oncol* **4**: 68–80

Bernier J, Thames HD, Smith CD and Horiot JC (1998) Tumour response, mucosal reactions and late effects after conventional and hyperfractionated radiotherapy. *Radiother Oncol* **47**: 137–143

Brock W, Baker FL and Peters LJ (1989) Radiosensitivity of human head and neck squamous cell carcinomas in primary culture and its potential as a predictive assay of tumor radiocurability. *Int J Radiat Biol* **56**: 751–760

Brock W, Baker FL, Wike JL, Sivon SL and Peters LJ (1990) Cellular radiosensitivity of primary head and neck squamous cell carcinomas and local tumour control. *Int J Radiat Oncol Biol Phys* **18**: 1283–1286

Broders A (1920) Squamous cell epithelioma of the lip. *J Am Med Assoc* **74**: 656–664

Bujko K, Skoczylas JZ, Burzykowski T, Hliniak A and Chmielewska E (1994) Is radiosensitivity of laryngeal mucosa similar to radiosensitivity of its malignant derivative squamous cell supraglottic cancer? *Radiother Oncol* **32**(suppl. 1): s87

Courtenay V (1984) A replenishable soft agar colony assay for human tumour sensitivity testing. *Recent Results Cancer Res* **94**: 17–34

Courtenay V, Selby PJ, Smith IE, Mills J and Peckham MJ (1978) Growth of human tumour cell colonies from biopsies using two soft-agar techniques. *Br J Cancer* **38**: 77–81

Dahl O, Horn A and Mella O (1994) Do acute side-effects during radiotherapy predict tumour response in rectal carcinoma? *Acta Oncol* **33**: 409

Dahlberg W, Little JB, Fletcher JA, Suit HD and Okunieff P (1993) Radiosensitivity in vitro of human soft tissue sarcoma cell lines and skin fibroblasts derived from the same patients. *Int J Radiat Biol* **63**: 191–198

Davidson SE, West CML, Roberts SA, Hendry JH and Hunter RD (1990) Radiosensitivity testing of primary cervical carcinoma: evaluation of intra- and inter-tumour heterogeneity. *Radiother Oncol* **18**: 349–356

Deacon J, Peckham MJ and Steel GG (1984) The responsiveness of human tumours and the initial slope of the cell survival curve. *Radiother Oncol* **2**: 317–323

Dische S, Saunders M, Barrett A, Harvey A, Gibson D and Parmar M (1997) A randomised multicentre trial of CHART vs conventional radiotherapy in head and neck cancer. *Radiother Oncol* **44**: 123–136

Eschwege F, Bourhis J, Girinski T, Lartigau E, Guichard M, Deblé D, Kepta L, Wilson GD and Luboinski B (1997) Predictive assays of radiation response in patients with head and neck squamous cell carcinoma: a review of the Institute Gustave Roussy experience. *Int J Radiat Biol Phys* **39**: 849–853

Fertil B and Malaise E (1981) Inherent cellular radiosensitivity as a basic concept for human tumour radiotherapy. *Int J Radiat Oncol Biol Phys* **7**: 621–629

Fertil B and Malaise E (1985) Intrinsic radiosensitivity of human cell lines is correlated with radioresponsiveness of human tumours: analysis of 101 published survival curves. *Int J Radiat Oncol Biol Phys* **11**: 1699–1707

Geara F, Peters LJ, Ang KK, Garden AS, Tucker SL, Levy LB and Brown BW (1996) Comparison between normal tissue reactions and local tumor control in head and neck cancer patients treated by definitive radiotherapy. *Int J Radiat Oncol Biol Phys* **35**: 455–462

Girinski T and Fertil B (1993) Predictive value of in vitro radiosensitivity parameters in head and neck cancers and cervical carcinomas: preliminary correlations with local control and overall survival. *Int J Radiat Oncol Biol Phys* **25**: 3–7

Girinski T, Bernheim A, Lubin R, Tavakoli-Razavi T, Baker F, Janot F, Wibault P, Cosset JM, Duvillard P, Duverger A and Fertil B (1994) In vitro parameters and treatment outcome in head and neck cancers treated with surgery and/or radiation: cell characterization and correlations with local control and overall survival. *Int J Radiat Oncol Biol Phys* **30**: 789–794

Hansen O, Overgaard J, Hansen H, Overgaard M, Høyer M, Jørgensen KE, Bastholt L and Berthelsen A (1997) Importance of overall treatment time for the outcome of radiotherapy of advanced head and neck carcinoma: dependency on tumor differentiation. *Radiother Oncol* **43**: 47–51

Haustermans K, Hofland I, Ramaekers M, Ivanyi D, Balm AJM, Geboes K, Lerut T, van der Schueren E and Begg AL (1996) Enrichment of tumor cells for cell kinetic analysis in human tumor biopsies using cytokeratin gating. *Radiother Oncol* **41**: 237–248

Hermanek P and Sobin L (eds) (1987) UICC's TNM Classification of Malignant Tumours. Head and Neck Tumours, p. 13–29. Springer-Verlag: Berlin

Kocagöncü K, Marangoni G, Cozzi-Fogliata A, Griffin S, Garavaglia G, Thum P and Bernier J (1994) Intrinsic radiosensitivity of head and neck carcinomas as predictive test for clinical tumour control: comparative analysis and critical assessment of technical reliability. *Radiat Oncol Invest* **2**: 177–184

Moll R, Franke WW, Schiller DL, Geiger B and Krepler R (1982) The catalog of human cytokeratins: patterns of expression in normal epithelia, tumours and cultured cells. *Cell* **31**: 11–24

Nordsmark M, Overgaard M and Overgaard J (1996) Pretreatment oxygenation predicts radiation response in advanced squamous cell carcinoma of the head and neck. *Radiother Oncol* **41**: 31–39

Peters LJ, Brock WA, Chapman JD and Wilson G (1988) Predictive assays of tumour radiocurability. *Am J Clin Oncol* **11**: 275–287

Schwartz J, Beckett M, Mustafi R, Vaughan A and Weichselbaum R (1992) Evaluation of different in vitro assays of inherent sensitivity as predictors of radiotherapy response. In: *Radiation Research: A Twentieth Century Perspective*, Dewey W, Edington M, Fry R, Hall E and Whitmore G (eds), p. 716–721. Academic Press: San Diego

Stausbøl-Grøn B, Nielsen OS, Bentzen SM and Overgaard J (1995) Selective assessment of in vitro radiosensitivity of tumour cells and fibroblasts from single tumour biopsies using immunocytochemical identification of colonies in the soft agar clonogenic assay. *Radiother Oncol* **37**: 87–99

Stausbøl-Grøn B, Bentzen SM and Overgaard J (1996) Predictive assays in radiotherapy. The Aarhus experience. In vitro radiosensitivity in SCC of the head and neck. *Radiother Oncol* **40** (suppl. 1): s68

Stausbøl-Grøn B, Bentzen SM and Overgaard J (1998) Characterisation and radiosensitivity of fibroblasts derived from squamous cell carcinomas of the head and neck, and the surrounding mucosa. *Acta Oncol* **37**: 697–700

Suo Z, Holm R and Nesland JM (1992) Squamous cell carcinomas, an immunohistochemical and ultrastructural study. *Anticancer Res* **12**: 2025–2032

Taghian A, Suit H, Pardo F, Gioioso D, Tomkinson K, Dubois W and Gerweck L (1992) In vitro intrinsic radiation sensitivity of glioblastoma multiforme. *Int J Radiat Oncol Biol Phys* **23**: 55–62

Tucker S, Turesson I and Thames HD (1992) Evidence for individual differences in the radiosensitivity of human skin. *Eur J Cancer* **28A**: 1783–1791

West CML (1995) Intrinsic radiosensitivity as a predictor of patient response to radiotherapy. *Br J Radiol* **68**: 827–837

West CML and Sutherland R (1986) A radiobiological comparison of human tumor soft-agar clonogenic assays. *Int J Cancer* **37**: 897–903

West CML, Davidson SE and Hunter RD (1989) Evaluation of surviving fraction at 2 Gy as a potential prognostic factor for the radiotherapy of carcinoma of the cervix. *Int J Radiat Biol* **56**: 761–765

West CML, Davidson SE, Roberts SA and Hunter RD (1993) Intrinsic radiosensitivity and prediction of patient response to radiotherapy for carcinoma of the cervix. *Br J Cancer* **68**: 819–823

West CML, Davidson SE, Roberts S and Hunter R (1997) The independence of intrinsic radiosensitivity as a prognostic factor for patient response to radiotherapy of carcinoma of the cervix. *Br J Cancer* **76**: 1184–1190

*© Cancer Research Campaign 1999*
